# Mutation in the Unrearranged *PML* Allele Confers Resistance to Arsenic Trioxide in Acute Promyelocytic Leukemia

**DOI:** 10.34133/research.0696

**Published:** 2025-05-06

**Authors:** Pei-Han Yu, Chen-Ying Zhu, Yuan-Yuan Kang, Hua Naranmandura, Chang Yang

**Affiliations:** ^1^ Department of Hematology of First Affiliated Hospital, Zhejiang University School of Medicine, Hangzhou 310058, China.; ^2^ Department of Public Health, Zhejiang University School of Medicine, Hangzhou 310058, China.; ^3^ Department of Pharmacology, Zhejiang University, Hangzhou 310058, China.; ^4^ Cancer Center, Zhejiang University, Hangzhou 310058, China.

## Abstract

Arsenic trioxide (ATO) is able to selectively target and degrade the disease-causing PML::RARα (P/R) oncoprotein in acute promyelocytic leukemia (APL) for curing the disease. However, some relapsed patients develop resistance to ATO due to mutations in the promyelocytic leukemia (PML) part of the *PML::RARα* fusion gene. A relapsed APL patient had shown resistance to ATO and chemotherapy and was identified to harbor a point mutation (A216V) in the unrearranged *PML* allele rather than the *PML::RARα* fusion gene. Here, we report that mutations in the unrearranged *PML* allele impede the ATO-induced destabilization and degradation of the wild-type P/R oncoprotein. Deletion of the coiled-coil domain in a PML mutant completely reversed wild-type P/R protein resistance to ATO by abolishing the interaction between PML and P/R proteins. Collectively, our findings reveal that a point mutation in the unrearranged *PML* allele can confer ATO resistance through a protein–protein interaction. Therefore, the unrearranged *PML* allele should also be screened for drug-resistant mutations in relapsed APL patients.

Acute promyelocytic leukemia (APL) is primarily caused by t(15;17) chromosomal translocation, which results in the fusion of promyelocytic leukemia (*PML*) gene with the retinoic acid receptor alpha (*RARα*) gene to generate the *PML::RARα* oncofusion gene [1,2]. The PML::RARα protein encoded by this oncofusion gene inhibits the physiological functions of normal PML as well as RARα proteins and abrogates the differentiation of myeloid cells at the promyelocyte stage [3]. Arsenic trioxide (ATO) has emerged as a first-line therapeutic agent for APL treatment in the clinic, owing to its effectiveness in targeting the disease-causing PML::RARα oncoprotein, resulting in the transition of the soluble PML::RARα protein into an insoluble form in the nuclear matrix, followed by SUMOylation and ubiquitination, ultimately leading to protein degradation [4]. Generally, the PML::RARα fusion protein consists of the RING (R), B-box1 (B1), B-box2 (B2), and coiled-coil (CC) domains termed as the RBCC domain in the PML portion [5]. Recent discoveries demonstrate that the binding of ATO to the tricysteine pocket formed by free Cys213 in the B2 domain is critical for its efficacy in degrading the PML::RARα protein [6]. Relapse and drug resistance after ATO treatment is a major challenge in the clinic for APL patients [7]. Mounting evidence indicates that the majority of drug-resistance cases arise from mutations (e.g., A216V and L218P) in the B2 domain of the PML::RARα oncoprotein, which can disrupt the tricysteine structure and impede ATO binding, resulting in resistance to ATO in the clinic [8].

Interestingly, mutations in the unrearranged *PML* allele, rather than classical mutations in the *PML::RARα* fusion gene, were observed in a few APL patients resistant to ATO treatment [9]. However, the intricate mechanisms have not yet been elucidated. Here, we report an APL patient that experienced 2 times relapse and exhibited ATO resistance. The treatment history is illustrated in Fig. 1A. However, genetic analysis showed that there was no mutation in the *PML::RARα* fusion gene; thus, we reasoned that a mutation might have occurred in the unrearranged *PML* allele (Fig. 1B). Indeed, an A216V mutation was identified in the unrearranged *PML* gene (Fig. 1C and D). Of note, primary APL blast cells obtained from this patient exhibited extreme resistance to ATO when compared with primary APL blast cells from an ATO-sensitive patient and APL cell line NB4 (Fig. 1E and F), which is consistent with clinical observations, suggesting that the mutant unrearranged *PML* allele might have conferred resistance to ATO treatment.

**Fig. 1.**
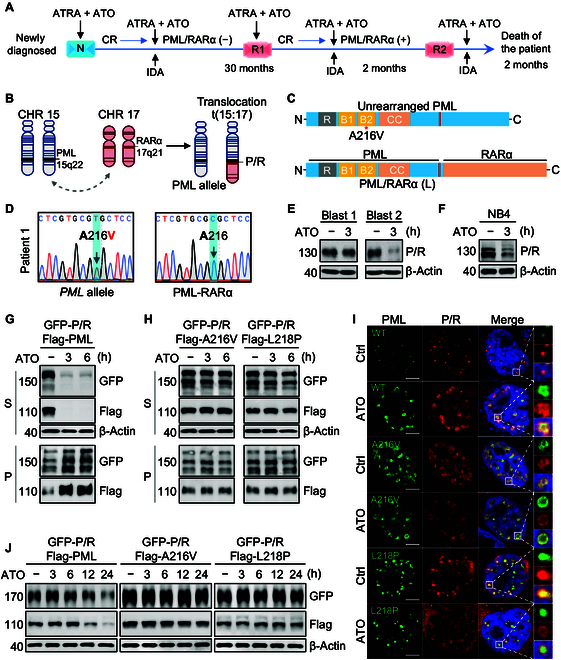
Promyelocytic leukemia (PML) mutants prevent arsenic trioxide (ATO)-induced destabilization, PML nuclear body (PML-NB) re-formation, and degradation of the wild-type (WT) PML::RARα (P/R)fusion protein. (A) The treatment history of a 47-year-old ATO-resistant acute promyelocytic leukemia (APL) patient. Initially, this patient was diagnosed as typical APL harboring the *PML::RARα* fusion gene and received ATO plus all-*trans*-retinoic acid (ATRA) treatment, achieving complete remission (CR) in January 2017. For consolidation, an ATO plus idarubicin (IDA) regimen was applied, and *PML::RARα* detection in the bone marrow (BM) turned negative. After 30 months, the patient experienced extramedullary relapse in the skin (R1). Consequently, after treatment with ATO plus ATRA and IDA as well as resection of the skin lesion, this patient achieved CR in January 2020. Two months later, the patient relapsed (R2) again and showed no response to ATO plus ATRA and IDA. Notably, there was a rapid increase in the level of P/R fusion within the bone marrow from 0.81% to 81%. Unfortunately, the patient died due to rapid disease progression. (B) APL is characterized by the reciprocal translocation t(15;17)(q24.1;q21.2), which fuses one *PML* gene on chromosome 15 and one *RARα* gene on chromosome 17 to generate the *PML::RARα* fusion gene while the *PML* allele is still present. (C) Domain structure of the unrearranged PML protein and P/R fusion proteins. Note the same RBCC domain (e.g., RING finger, B-box, and coiled-coil) in the N-terminus but different in the C-terminus. (D) Genetic analysis of the unrearranged *PML* allele and the *PML::RARα* fusion gene in this ATO-resistant patient. (E and F) Primary APL blast cells were obtained from the 47-year-old ATO-resistant patient (blast 1) and an ATO-sensitive patient (blast 2). Subsequently, primary blast cells were exposed to ATO (1 μM) for 3 h, and the P/R fusion protein degradation was analyzed by western blotting. NB4 cells were used as positive control. (G and H) Green fluorescent protein (GFP)-labeled WT P/R plasmid was transiently cotransfected with Flag-tagged WT PML, A216V-PML mutant, and L218P-PML mutant in PML^−/−^ HeLa for 24 h and then exposed to ATO (1 μM) for 3 and 6 h. Solubility changes in the P/R fusion protein and PML proteins were detected by western blotting. (I) Changes in PML-NBs were determined by confocal microscopy. (J) PML^−/−^ HeLa cells coexpressing WT P/R with WT PML, A216V-PML mutant, and L218P-PML mutant were exposed to ATO (1 μM) for 3, 6, 12, and 24 h to determine protein degradation. Total proteins were extracted from cells by a urea buffer as described in Supplementary Materials and Methods. Green fluorescence indicates the PML protein, red fluorescence indicates the P/R fusion protein, and blue fluorescence (4′,6-diamidino-2-phenylindole [DAPI]) indicates the nucleus. S indicates supernatant; P indicates insoluble pellet. The scale bar is 5 μm. RARα, retinoic acid receptor alpha.

To test our hypothesis, we compared the sensitivity of the wild-type (WT) PML protein, A216V-PML and L218P-PML mutants, and the WT PML::RARα fusion protein to ATO. Results showed that ATO could effectively induce transition of WT PML as well as the PML::RARα protein from a soluble state (S) to an insoluble state (P) but had no effect on A216V-PML and L218P-PML mutants (Fig. S1A and B). Furthermore, confocal microscopy showed that PML mutants formed giant PML nuclear bodies (PML-NBs) accompanied by a few small PML-NBs, which is distinct from the morphology of WT PML-NBs, while the PML::RARα fusion protein was mainly diffused in the nucleus. ATO treatment enhanced the size of PML-NBs formed by WT PML and re-formed PML-NBs for the WT PML::RARα fusion protein, but no changes were observed in 2 PML mutants (Fig. S1C to F). These findings indicate that PML mutants are indeed resistant to ATO treatment.

Notably, the PML::RARα oncoprotein and unrearranged PML protein commonly coexist in APL patients [10]. To reveal whether unrearranged PML mutants could alter the sensitivity of the WT PML::RARα protein to ATO treatment, we coexpressed WT PML::RARα with WT PML or PML mutants (A216V and L218P) in PML^−/−^ HeLa cells to mimic the situation in APL patients who are sensitive or resistant to ATO. The results showed that ATO is capable of inducing WT PML::RARα protein transition from a soluble state to an insoluble state (S to P) in the presence of the WT PML protein (Fig. 1G and Fig. S2A). Surprisingly, ATO lost the ability to induce destabilization of the WT PML::RARα protein in the presence of the A216V-PML mutant and/or the L218P-PML mutant (Fig. 1H and Fig. S2B and C), suggesting that PML mutants alter the sensitivity of WT PML::RARα to ATO treatment. Conversely, when coexpressing WT PML with an ATO-resistant A216V-PML::RARα mutant, ATO is also unable to induce destabilization and degradation of the WT PML protein (Fig. S2D), demonstrating that the mutant PML::RARα protein also alters the ATO sensitivity for the WT PML protein. Interestingly, a decrease in A216V-PML protein level resulted in markedly heightened sensitivity of the WT PML::RARα protein toward ATO (Fig. S2B). However, even a minimal amount of the L218P-PML mutant was sufficient to maintain the resistance of WT PML::RARα to ATO (Fig. S2C), implying that A216V-PML and L218P-PML mutant proteins have distinct ability to influence the sensitivity of the WT PML::RARα protein to ATO treatment. Moreover, ATO also accelerated the re-formation of PML-NBs when WT PML::RARα was coexpressed with WT PML rather than with mutant PMLs (Fig. 1I). Likewise, immunoblotting showed that ATO effectively degraded the WT PML::RARα protein in a time-dependent manner in the presence of the WT PML protein, but not in the presence of A216V-PML and L218P-PML mutant proteins (Fig. 1J). These findings demonstrate that PML mutants impede ATO-induced destabilization and degradation of the WT PML::RARα protein.

Posttranslational modifications play pivotal regulatory roles in PML::RARα fusion protein degradation induced by ATO [11]. SUMOylation of WT PML::RARα was dramatically increased in the presence of the WT PML protein rather than mutant PMLs under ATO treatment (Fig. 2A and Fig. S3A to F). Similarly, confocal images showed that small ubiquitin like modifier 1 (SUMO-1) colocalized with WT PML::RARα and the WT PML protein after ATO treatment, but not in WT PML::RARα with PML mutant proteins (Fig. 2B and Fig. S3G). Moreover, only the WT PML::RARα fusion protein was able to recruit the functional partner speckled protein 100 (SP100) in the presence of the WT PML protein, indicating that mutant PMLs indeed impair the recruitment of functional partner proteins (e.g., SUMO-1 and SP100) to the PML::RARα fusion protein under ATO treatment (Fig. 2C).

In the light of the above findings, we hypothesized that the unrearranged PML and PML::RARα proteins could functionally interact with each other. Here, confocal microscopy showed that PML::RARα expression led to the structural disintegration of normal PML-NBs, resulting in a characteristic microdispersed distribution pattern of the PML protein throughout the nucleoplasm (Fig. 1I). Similarly, PML-NBs formed by A216V and/or L218P mutants could also be disrupted by the PML::RARα protein (Fig. 1I), but each mutant PML protein was found to be colocalized with the PML::RARα protein. More interestingly, filaments like the structure formed by an artificial PML mutant L218Y has no response to ATO treatment, while it can be disassembled upon coexpression with the PML::RARα protein (Fig. S4A and B). These findings suggest potential interaction between the PML::RARα and PML proteins.

The interactions between PML::RARα and WT or mutant PML proteins were further determined by immunoprecipitation. The results showed that A216V-PML and L218P-PML mutants exhibited higher binding affinity to the PML::RARα protein than WT PML (Fig. 2D). Surprisingly, protein–protein interactions were further enhanced by ATO between PML::RARα and PML mutants, but not in the WT PML protein (Fig. S5A). This appears to be associated with the increased dynamics of mutant PML proteins as previously reported [6]. Generally, the RBCC domain of PML has been established as an essential structure for PML-NB formation by inducing the oligomerization of PML proteins [12–15]. To further clarify the role of each domain in PML protein oligomerization, we constructed domain truncated mutants (*Δ*RING, *Δ*B1, *Δ*B2, and *Δ*CC) in WT PML and examined the changes in multimerization for the PML protein by native polyacrylamide gel electrophoresis (Fig. 2E). Surprisingly, deletion of the B2 domain only disrupted the polymerization of the PML protein, whereas deletion of the CC domain completely abolished the homo-dimerization of the PML protein (Fig. 2F). Thus, we reasoned that the unrearranged PML protein and PML::RARα protein might interact with each other through CC domains. In order to test this hypothesis, the WT PML::RARα protein was coexpressed with the truncated mutant PMLs (*Δ*RING, *Δ*B1, *Δ*B2, and *Δ*CC), respectively. Interestingly, only truncation of the CC domain rather than other domains in the A216V-PML mutant abolished the protein–protein interactions (Fig. 2G and Fig. S5B). Moreover, similar results were also obtained when the CC domain was truncated in WT PML::RARα or PML proteins (Fig. S5C and D), indicating that the unrearranged PML protein and PML::RARα protein form a heterodimer through the CC domain. Excitingly, the truncation of the CC domain in mutant PML restored the sensitivity of the WT PML::RARα protein to ATO (Fig. 2G and H and Fig. S5E), suggesting that unrearranged PML mutant caused ATO resistance is mediated by interaction between WT PML::RARα and PML mutant proteins through the CC domain.

Here, we report an ATO-resistant APL patient harboring A216V mutation in the unrearranged *PML* allele rather than in the *PML::RARα* fusion gene. We found that ATO lost the ability to induce WT PML::RARα protein destabilization and degradation, as well as re-formation of SUMOylated PML-NBs in the presence of unrearranged PML mutant proteins, indicating that unrearranged PML mutants confer resistance of the WT PML::RARα fusion protein to ATO treatment. Notably, deletion of the CC domain, rather than the RING, B1, and B2 domains, in PML mutants restored the sensitivity of the WT PML::RARα protein to ATO, demonstrating that PML mutants can alter the ATO sensitivity of the WT PML::RARα protein through CC-domain-mediated protein–protein interactions. Our findings indicate that the response of the unrearranged PML protein and WT PML::RARα protein to ATO treatment is not independent when they coexist in APL cells, which may explain why the APL patients harboring a mutation in the unrearranged *PML* allele exhibit ATO resistance. Therefore, we suggest that the unrearranged *PML* allele should be analyzed for screening mutations, especially when no mutations are observed in the *PML::RARα* gene in ATO-resistant APL patients.

**Fig. 2.**
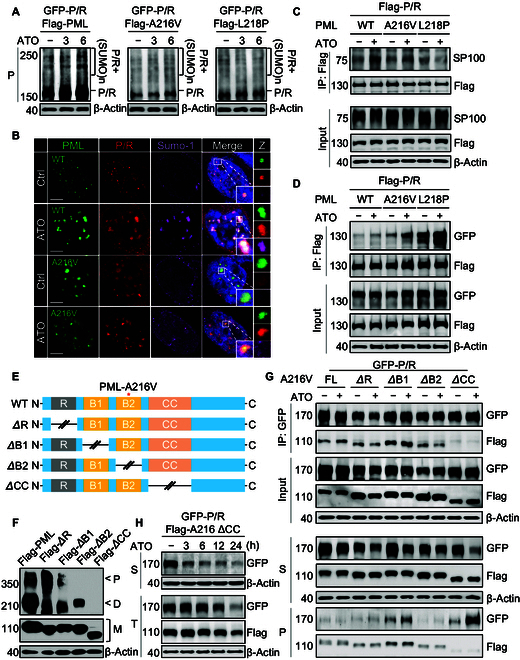
Unrearranged PML mutants alter the sensitivity of the WT P/R fusion protein to ATO through protein–protein interaction mediated by the coiled-coil (CC) domain. (A) A GFP-labeled WT P/R plasmid was transiently cotransfected with Flag-tagged WT PML, A216V-PML mutant, and L218P-PML mutant into PML^−/−^HeLa cells for 24 h and then exposed to 1 μM ATO for 3 and 6 h. SUMOylation of PML proteins was determined by western blotting with PML antibody. (B) Colocalization of PML-NBs with small ubiquitin like modifier 1 (SUMO-1) in cells was detected by confocal microscopy. (C) Interactions between speckled protein 100 (SP100) and Flag-P/R in the presence of each WT PML, A216V-PML mutant, and L218P-PML mutant were determined by co-immunoprecipitation (co-IP) in PML^−/−^ HeLa cells after treatment with ATO (1 μM) for 6 h. (D) PML^−/−^ HeLa cells coexpressing GFP-P/R with each Flag-PML (i.e., WT, A216V, and L218P) were exposed to ATO at 1 μM for 6 h. Interaction of PML proteins with the P/R fusion protein was determined by co-IP. (E) Schematic diagram of the RING (*Δ*R), B-box1 (*Δ*B1), B-box2 (*Δ*B2), and coiled-coil (*Δ*CC) domain truncated PMLs. (F) Changes in PML multimerization in truncated PML proteins were determined by native gel electrophoresis. M indicates monomer, D indicates dimer, and P indicates polymer. (G) Interactions between GFP-P/R and each of *Δ*R, *Δ*B1, and *Δ*B2 as well as *Δ*CC-A216V-PML mutants in PML^−/−^ HeLa were analyzed by co-IP after treatment with ATO (1 μM) for 6 h. Solubility changes of the P/R fusion protein and PML protein were determined by western blotting. (H) PML^−/−^ HeLa cells expressing GFP-P/R and CC truncated Flag-PML-A216V were exposed to ATO (1 μM) for 3, 6, 12, and 24 h, and then the changes in the total protein levels of the GFP-P/R fusion protein and *Δ*CC-A216V-PML protein were determined. Green fluorescence indicates PML protein, red fluorescence indicates P/R fusion protein, purple fluorescence indicates SUMO-1, and blue fluorescence (DAPI) indicates the nucleus. S indicates supernatant, P indicates insoluble pellet, and T indicates total protein. The scale bar is 5 μm.

## Data Availability

All data generated or analyzed during this study are included in this published article and its Supplementary Materials file.
