# Supplementary material for: Mutation in the Unrearranged PML Allele Confers Resistance to Arsenic Trioxide in Acute Promyelocytic Leukemia
Source: Research (Wash D C). 2025 May 6;8:0696. doi: 10.34133/research.0696 (PMC12053449; doi:10.34133/research.0696)
Supplement: Supplementary 1 — Materials and Methods Figs. S1 to S5 [file research.0696.f1.pdf]

Supplement Online Material for

Mutation in the Unrearranged PML Allele Confers Resistance to Arsenic Trioxide in Acute Promyelocytic Leukemia

Pei-Han Yu[a,b,c§], Chen-Ying Zhu[a,b§], Yuan-Yuan Kang[a,b,c], Hua Naranmandura[a,b,c,d*], Chang Yang[a,b*]

This PDF file includes Materials and Methods, Supplementary Figure Legends for Fig.S1-S5 and Supplementary FigureS1-S5

*To whom correspondence to:
Dr. Chang Yang,
Department of Public Health, School of Medicine, Zhejiang University,
Hangzhou, Zhejiang, 310058, China,
E-mail: yangchang85@zju.edu.cn
Dr. Hua Naranmandura
Department of Toxicology, School of Medicine and Public Health, Zhejiang University,
Hangzhou, Zhejiang, 310058, China
Fax/Phone: (86) 571-8820-6736
E-mail: narenman@zju.edu.cn
§ These authors contributed equally to this work

Materials and Methods

Human specimens

The study was approved by the Ethics Committee of the Zhejiang University School of Medicine (#2018-023) and was conducted in accordance with the Declaration of Helsinki. Primary human APL blasts were obtained from apheresis products or bone marrow of patients with APL who gave written consent for sample procurement.

Cell Lines and Primary Cultures

HeLa cell line was purchased from the Cell Bank of Chinese Academy of Sciences. Following receipt, cells were grown and frozen as a seed stock as they were available. HeLa cell line was authenticated using DNA fingerprinting (variable number of tandem repeats), confirmed that no cross-contamination occurred during this study, and tested for Mycoplasma contamination once in a year. Cells were cultured and maintained in DMEM (BasalMedia, L110KJ) with 10% fetal bovine serum (Gibco, 10270-106). The NB4 cell line was purchased from Creative Bioarray. NB4 cells were cultured and maintained in RPMI-1640 medium (Gibco, 12800-017), the culture media were supplemented with 10% fetal bovine serum (Gibco, 10270-106). All the culture media were supplemented with 100 U/mL penicillin, and 100 μg/mL streptomycin. Cells were kept at 37°C in 5% CO2 atmosphere.

Reagents

The chemicals and reagents used in this experiment were of analytical grade standard. Water was sourced from a Millipore system (Bedford, MA, USA). Sodium arsenate (iAs^{III}) was acquired from Wako Pure Chemical Industries, Ltd. (Osaka, Japan). Hieff Trans® Suspension Cell-Free Liposomal Transfection Reagent was purchased from Yeasen Company (Shanghai, China).

Antibodies

Rabbit anti-human PML monoclonal (EPR16792) antibody and Mouse anti-SP100 polyclonal antibody were purchased from Abcam (Cambridge, UK). Rabbit anti-β-actin monoclonal (13E5) antibody and anti-SUMO-1 monoclonal (C9H1) antibody were purchased from Cell Signaling Technology (Danvers, MA). Mouse anti-FLAG monoclonal (M2) antibody and anti-GFP monoclonal (1E10H7) antibody were purchased from Sigma (St. Louis, MO, USA) and Proteintech (Rosemont, USA) respectively.

Primers for Detection of Mutations in *PML* allele and *PML:: RARα* fusion genes

To accurately detect mutations in *PML::RARα* fusion genes and *PML* alleles, we designed specific primers for both *PML* alleles and *PML:: RARα* fusion genes. The sequence and location of primers were provided as follows:

PML-Exon2-F: CACACCAGTGGTTCCTCAAG;

PML-Exon7-R: CGGCATCTGAGTCTTCCGAG

P/R-Exon3-R: TGCTGCTCTGGGTCTCAATG.

Plasmid construction

PML gene was amplified from cDNA of HeLa cells with forward primer and reverse primers. PML Forward: CGGATCCATGGAGCCTGCACCCGCCCGAT; PML Reverse: CCTCGAGCTAAATTAGAAAGGGGTGGGGG. FLAG-tagged PML and PML-RARα were inserted in PCMV-Tag2B vector, while GFP-labeled PML and PML-RARα were inserted in pEGFP-N1 vector (Sigma). These plasmids were transiently transfected into PML$^{-/-}$ HeLa cells in FBS-free medium which was removed by complement medium after 6h. Liposomal transfection reagent was used for the purpose of transfection of plasmids into PML$^{-/-}$ HeLa cells, according to the manufacturer's instructions.

Construction of PML Knockout (PML-/-) HeLa Cells by CRISPR-Cas9

PML−/− HeLa cells were generated from wild type HeLa cells by CRISPR-Cas9. The online CRISPR Design Tool (http:/tools.genome-engineering.org) were used to take an input target genomic DNA sequence and identify the suitable guide sequence pairs (Forward: CACCGCAATCTGCCGGT ACACCGAC; Reverse: AAACGTCGGTGTACCGGCAGATTGC). The sgRNA expression construct was generated by PCR amplification and cloned into pSpCas9(BB) vector for co-expression with Cas9 in wild type HeLa cells. Partial of the transfected cells were assayed for sgRNA functional validation by Surveyor nuclease assay. The genome of the cells was extracted, and the target DNA sequence was amplified by Surveyor primers (Forward: GAGGAGTTCCAGTTTCTG; Reverse: GATCTTTG CTCCTCTCG) through Surveyor PCR. After purification, PCRs were annealed for DNA heteroduplex formation which was further digested by mismatched enzyme (T7E1) and visualized on agarose gel. The sgRNA was effective if main target band became weak and sheared into two shallow bands. Cells transfected with the effective sgRNA were selected and cultured with complement medium containing 1 μg/ml puromycin. Several monoclonal cell clusters were formed and selected under the microscope, and then cultured in complement medium. Genomic sequence of each monoclonal cell cluster was determined by sequencing and the expression of endogenous PML was detected by western blot. PML-/- cell line was the one with frameshift mutation at targeted site and no detectable PML protein.

Protein Extraction and Western Blot Analysis

Cells were washed twice with D-Hank's solution, followed by lysis of cell pellets using RIPA lysis buffer (50 mmol/L Tris, 150 mmol/L NaCl, 1% NP-40, 0.5% sodium deoxycholate, 0.1% SDS, pH 7.5, 0.2 mmol/L PMSF, and a complete mini protease inhibitor tablet) to obtain whole-cell lysates. Samples were incubated on ice for 15

minutes with vortexes in 5-minute intervals and centrifuged for 30 minutes at 4°C, 13,000 rpm to obtain the supernatant (S) for Western blot analysis. The pellet (P) was washed twice with PBS, centrifuged for 10 minutes at 4°C, and lysed in LDS buffer (1 × TBS, 10% glycerol, 0.015% EDTA, 50 mmol/L DTT, and 2% SDS) by boiling for 10 minutes at 95°C. Protein concentrations were measured by BCA Protein Quantification Kit (Yeasen Biotech, 20201ES76). Each protein sample (20 μg) was resolved by 8% SDS-PAGE and blotted onto PVDF membranes. The membranes were blocked with non-fat milk and incubated overnight with different antibodies at 4°C, followed by incubation with HRP-labeled secondary antibodies for 1 hour at room temperature. Protein bands were visualized by enhanced chemiluminescence (Biological Industries; 20–500–120).

Immunoprecipitation

Cells were planted in 10-cm culture dishes and transfected with each indicated plasmid. 24 hours after transfection, cells were treated as indicated and collected in ice-cold PBS. Each sample was lysed on ice by sonication in 900 μL IP buffer (50 mmol/L Tris-HCl pH7.5, 10% glycerol, 150 mmol/L NaCl, 2 mmol/L EDTA, 0.5% NP-40, and 1 mmol/L PMSF plus protease inhibitors). Immunoprecipitation experiments were performed using protein A/G PLUS-Agarose Immunoprecipitation Reagent (CST, sc-2003) according to the manufacturer's instructions.

Immunofluorescence Microscopy

HeLa cells were grown in culture wells containing glass disks and transferred onto glass slides following indicated treatments. Slides were washed twice with PBS, fixed in 4% paraformaldehyde for 30 minutes, and permeabilized with 0.1% Triton X-100 for 30 minutes. Then slides were blocked with 2% BSA in PBS, followed by incubation with primary antibodies overnight at 4°C. The next day, slides were washed 3 times with

PBS and incubated with fluorescent-labeled secondary antibodies at room temperature for 4 hours. Slides were mounted using DAPI Fluoromount-G (SouthernBiotech, 0100-20) and stored in the dark at 4℃. The fluorescent signals were visualized under a Leica STELLARIS8 confocal microscope.

Supplementary Figure Legends

Fig.S1. Effect of ATO on Solubility and Morphological Changes in Wild-Type PML, PML Mutants, and PML/RARα Fusion Proteins

(**A**) Wild-type (WT) PML, A216V-PML mutant, and L218P-PML mutant, as well as (**B**) WT-PML/RARα (P/R) plasmids were transiently transfected into $PML^{-/-}$HeLa cells for 24h, and then exposed to 1μM ATO for 3, 6, 12h. Protein solubility changes were determined by western blotting. Formation of PML nuclear bodies (PML-NBs) in $PML^{-/-}$HeLa cells expressing WT-PML (**C**), A216V-PML mutant (**D**), L218P-PML mutant (**E**), and WT-P/R (**F**) was determined by confocal microscopy with or without ATO treatment. Green fluorescence indicates PML or P/R fusion protein. Blue fluorescence (DAPI) indicates the nucleus. S indicates supernatant; P indicates insoluble pellet; Scale bar is 5 μm.

Fig.S2. PML Mutants Prevent Destabilization of Wild-type (WT) PML/RARα Fusion Protein by ATO Treatment

GFP-labeled WT-P/R plasmid was transiently co-transfected with Flag-tagged WT-PML (**A**), A216V-PML mutant (**B**) and L218P-PML mutant (**C**) respectively into $PML^{-/-}$HeLa cells in varying plasmid ratios, and then exposed to 1μM ATO for 6h. Likewise, (**D**) GFP-labeled WT-PML plasmid was transiently co-transfected with Flag-tagged WT-P/R, A216V-P/R mutant and L218P-P/R mutant in $PML^{-/-}$ HeLa cells for 24h, and then exposed to 1μM ATO for 3 and 6h. Protein solubility changes for P/R fusion protein and PML proteins were detected by western blotting. S indicates supernatant; P indicates insoluble pellet.

Fig.S3. Mutations in PML and PML/RARα Fusion Protein Prevents their SUMOylation induced by ATO Treatment

Flag-tagged WT-PML (**A**), A216V-PML mutant (**B**), L218P-PML mutant (**C**), WT-P/R

(**D**), A216V-P/R mutant (**E**), and L218P-P/R mutant (**F**) plasmids were transiently transfected into PML$^{-/-}$HeLa cells for 24h, and then exposed to ATO (1μM) for 3 and 6h. SUMOylation of PML and P/R proteins was determined by western blotting. On the other hand, PML$^{-/-}$ HeLa cells co-expressing L218P-PML mutant with WT-P/R were treated with ATO (1μM) for 6h, and P/R protein SUMOylation was determined by confocal microscopy (**G**). Green fluorescence indicates L218P-PML mutant, red fluorescence indicates P/R fusion protein, purple fluorescence indicates SUMO-1, and blue fluorescence (DAPI) indicates the nucleus; P indicates insoluble pellet; Scale bar is 5 μm.

Fig.S4. PML/RARα Fusion Protein Alters the Morphology of PML-NBs formed by PML mutants

(**A**) Formation of PML-NBs by L218Y-PML mutant protein was determined by confocal microscopy with or without ATO treatment, and (**B**) changes in PML-NBs formed by L218Y-PML mutant protein in the presence of P/R was determined by confocal microscopy. Green fluorescence indicates mutants L218Y-PML mutant protein, red fluorescence indicates P/R fusion protein, blue fluorescence (DAPI) indicates the nucleus; Scale bar is 5 μm.

Fig.S5. Deletion of CC domain Abolishes Protein-Protein Interaction Between Unrearranged PML and PML/RARα fusion proteins

(**A**) PML$^{-/-}$ HeLa cells co-expressing Flag-PML (WT) with GFP-PMLs (i.e., WT, A216V or L218P) respectively were exposed to ATO (1μM) for 6h. Interactions between Flag-PML and GFP-PML proteins were determined by co-IP. Moreover, interactions between GFP-P/R and unrearranged PML CC truncation mutants (**B**), as well as GFP-P/R CC truncation mutants and unrearranged PML proteins (**C**) were analyzed in PML$^{-/-}$ HeLa cells by co-IP after treatment with ATO (1μM) for 6h. (**D**)

Interaction between GFP-P/R and each truncation mutant (e.g., ΔR, ΔB1, ΔB2 and ΔCC) of WT-PML were determined in $PML^{-/-}$ HeLa cells after treatment with ATO (1μM) for 6h. (**E**) Protein solubility shift of P/R and PML proteins was determined by western blotting in $PML^{-/-}$ HeLa cells co-expressing GFP-P/R with each of full length (FL)-PML, ΔR, ΔB1, ΔB2 and ΔCC-PMLs after treatment with ATO (1μM) for 6h. S indicates supernatant; P indicates insoluble pellet.

Fig.S1

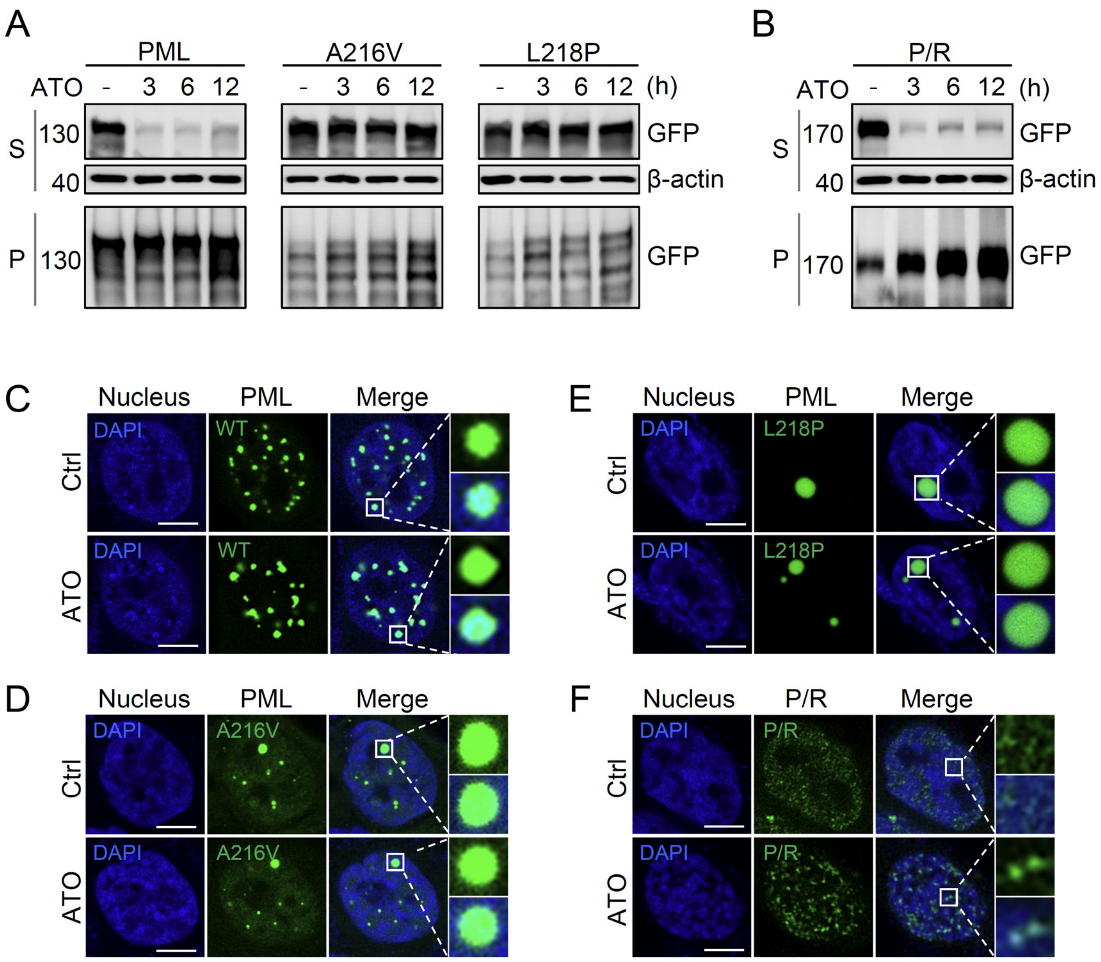
A
PML
A216V
L218P
ATO - 3 6 12 (h)
S
130
40
GFP
β-actin
P
130
GFP
B
P/R
ATO - 3 6 12 (h)
S
170
40
GFP
β-actin
P
170
GFP
C
Nucleus PML Merge
Ctrl
ATO
DAPI
WT
D
Nucleus PML Merge
DAPI
A216V
E
Nucleus PML Merge
DAPI
L218P
F
Nucleus P/R Merge
DAPI
P/R

Fig.S2

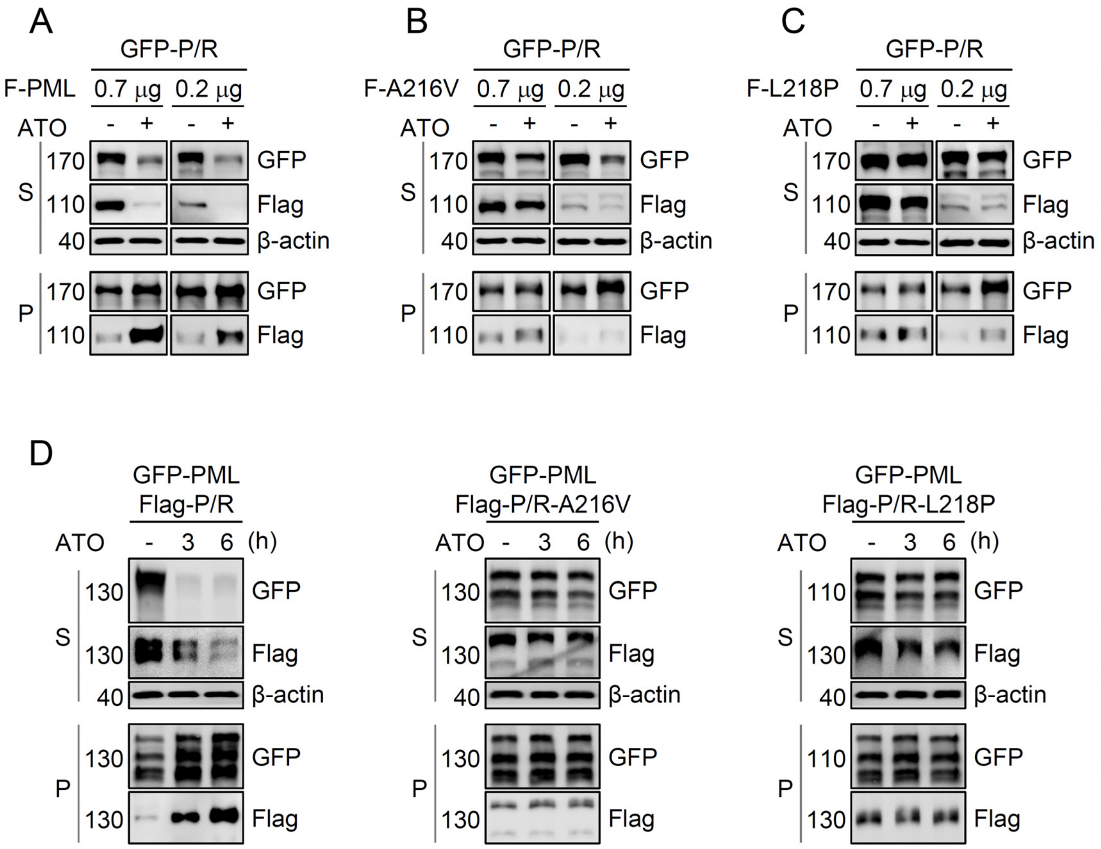
A
GFP-P/R
F-PML 0.7 μg 0.2 μg
ATO - + - +
S
170 GFP
110 Flag
40 β-actin
P
170 GFP
110 Flag
B
GFP-P/R
F-A216V 0.7 μg 0.2 μg
ATO - + - +
S
170 GFP
110 Flag
40 β-actin
P
170 GFP
110 Flag
C
GFP-P/R
F-L218P 0.7 μg 0.2 μg
ATO - + - +
S
170 GFP
110 Flag
40 β-actin
P
170 GFP
110 Flag
D
GFP-PML
Flag-P/R
ATO - 3 6 (h)
S
130 GFP
130 Flag
40 β-actin
P
130 GFP
130 Flag
GFP-PML
Flag-P/R-A216V
ATO - 3 6 (h)
S
130 GFP
130 Flag
40 β-actin
P
130 GFP
130 Flag
GFP-PML
Flag-P/R-L218P
ATO - 3 6 (h)
S
110 GFP
130 Flag
40 β-actin
P
110 GFP
130 Flag

Fig.S3

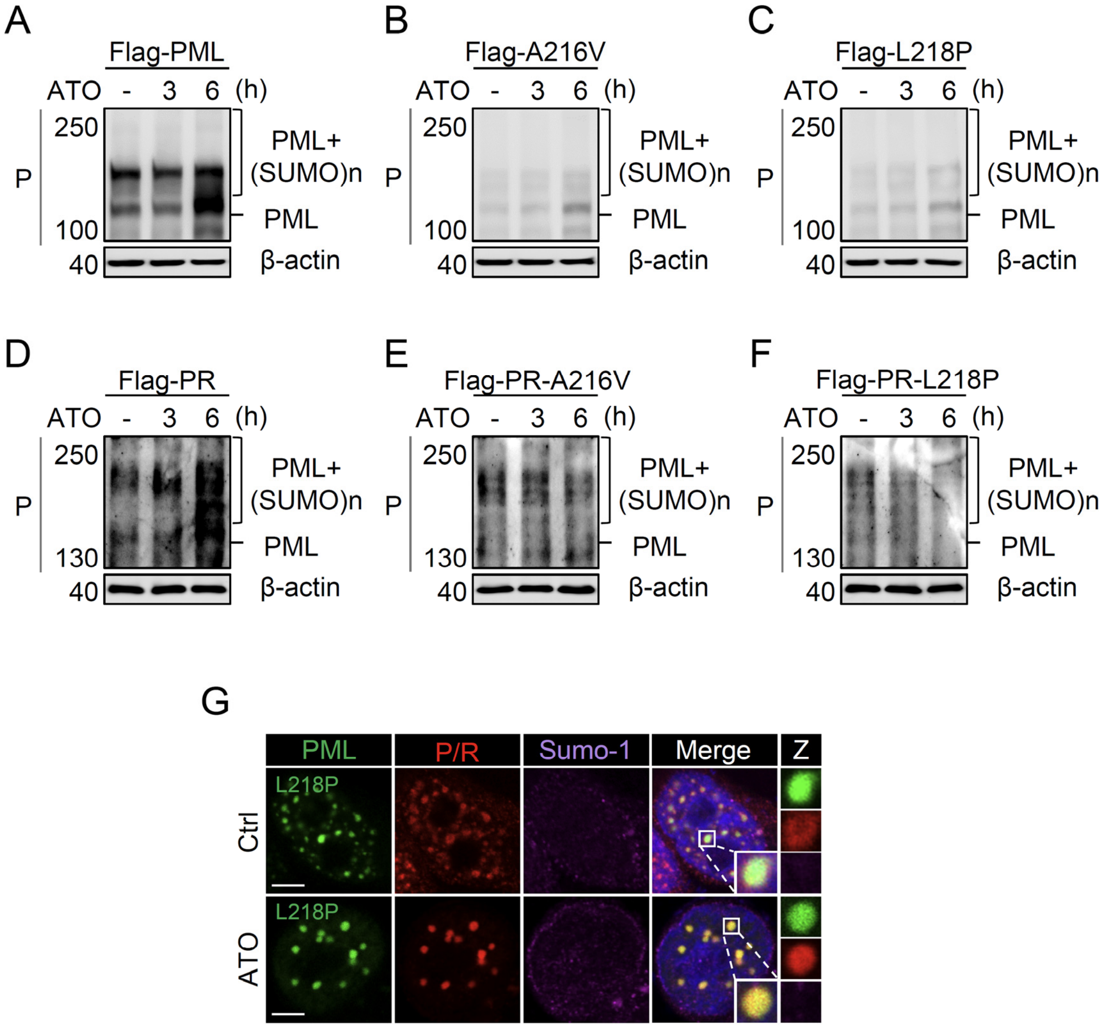
A
Flag-PML
ATO - 3 6 (h)
250
P
100
40
PML+
(SUMO)n
PML
β-actin
B
Flag-A216V
C
Flag-L218P
D
Flag-PR
130
E
Flag-PR-A216V
F
Flag-PR-L218P
G
PML
P/R
Sumo-1
Merge
Z
L218P
Ctrl
ATO

Fig.S4

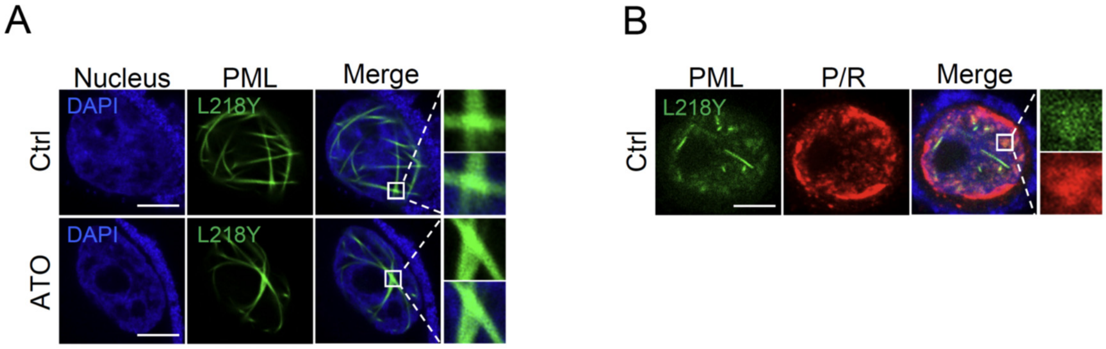

A
Nucleus
PML
Merge
Ctrl
ATO
DAPI
L218Y
DAPI
L218Y
B
PML
P/R
Merge
Ctrl
L218Y

Fig.S5

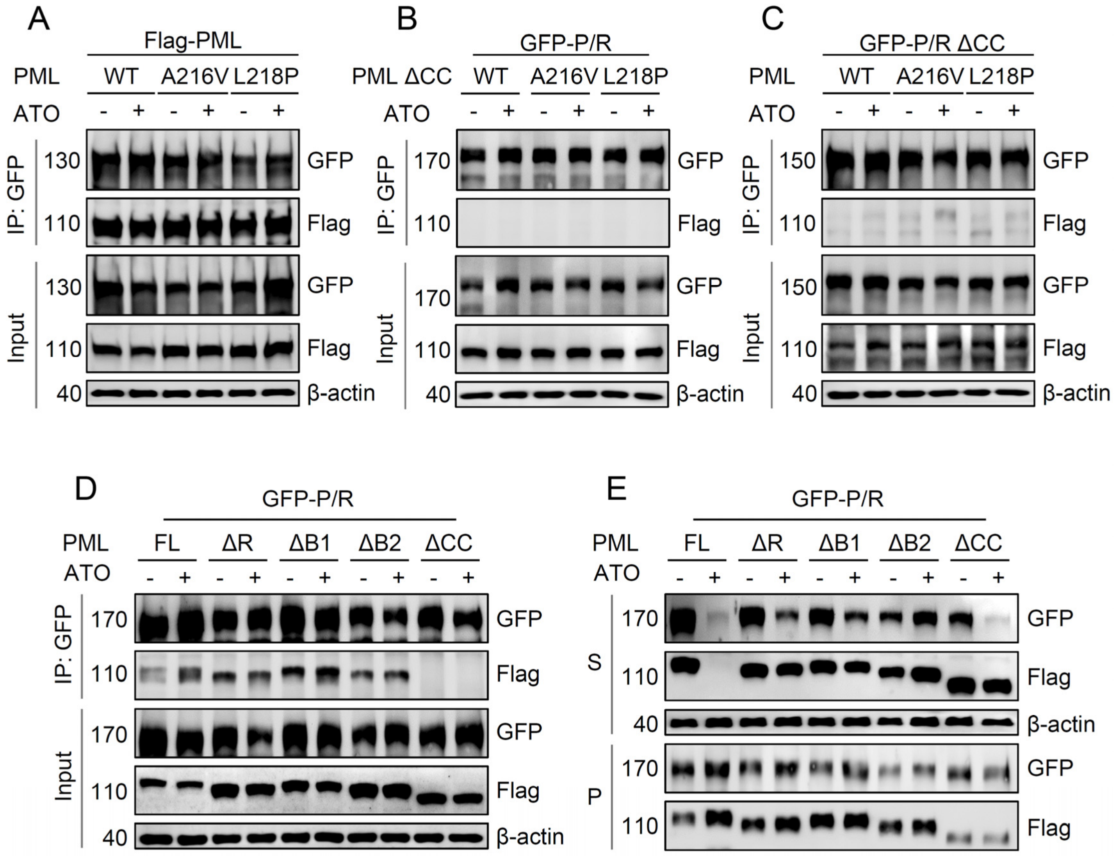

A
Flag-PML
PML WT A216V L218P
ATO - + - + - +
IP: GFP
130 GFP
110 Flag
Input
130 GFP
110 Flag
40 β-actin
B
GFP-P/R
PML ΔCC WT A216V L218P
ATO - + - + - +
IP: GFP
170 GFP
110 Flag
Input
170 GFP
110 Flag
40 β-actin
C
GFP-P/R ΔCC
PML WT A216V L218P
ATO - + - + - +
IP: GFP
150 GFP
110 Flag
Input
150 GFP
110 Flag
40 β-actin
D
GFP-P/R
PML FL ΔR ΔB1 ΔB2 ΔCC
ATO - + - + - + - + - +
IP: GFP
170 GFP
110 Flag
Input
170 GFP
110 Flag
40 β-actin
E
GFP-P/R
PML FL ΔR ΔB1 ΔB2 ΔCC
ATO - + - + - + - + - +
S
170 GFP
110 Flag
40 β-actin
P
170 GFP
110 Flag
